# Current Perspectives in NSAID-Induced Gastropathy

**DOI:** 10.1155/2013/258209

**Published:** 2013-03-12

**Authors:** Mau Sinha, Lovely Gautam, Prakash Kumar Shukla, Punit Kaur, Sujata Sharma, Tej P. Singh

**Affiliations:** Department of Biophysics, All India Institute of Medical Sciences, Ansari Nagar, New Delhi 110 029, India

## Abstract

Nonsteroidal anti-inflammatory drugs (NSAIDs) are the most highly prescribed drugs in the world. Their analgesic, anti-inflammatory, and antipyretic actions may be beneficial; however, they are associated with severe side effects including gastrointestinal injury and peptic ulceration. Though several approaches for limiting these side effects have been adopted, like the use of COX-2 specific drugs, comedication of acid suppressants like proton pump inhibitors and prostaglandin analogs, these alternatives have limitations in terms of efficacy and side effects. In this paper, the mechanism of action of NSAIDs and their critical gastrointestinal complications have been reviewed. This paper also provides the information on different preventive measures prescribed to minimize such adverse effects and analyses the new suggested strategies for development of novel drugs to maintain the anti-inflammatory functions of NSAIDs along with effective gastrointestinal protection.

## 1. Introduction

 Nonsteroidal anti-inflammatory drugs (NSAIDs) are the most well recognized drugs worldwide for the treatment of pain, inflammation, and fever [1–4]. NSAIDs are commonly administered for treatment against inflammatory diseases, rheumatoid arthritis, osteoarthritis, dysmenorrhea, and ischemic cerebrovascular disorders [5]. Use of these drugs in certain types of cancer treatment has also been reported recently [6, 7]. These drugs inhibit prostaglandin biosynthesis and produce their therapeutic effects [8]. However, long-term administration of NSAIDs causes adverse gastrointestinal (GI) symptoms including mucosal lesions, bleeding, peptic ulcer, and inflammation in intestine leading to perforation, strictures in small and large intestines, leading to chronic problems [9–11]. Some of the adverse effects of NSAIDs may be asymptotic, but in many cases there are reports of life-threatening incidents [10]. 

 Such rampant use of NSAIDs requires a focused approach to avoid the possible side effects arising from their use. In this regard, several prevention methods have been used. These are based on usage of a new class of NSAIDs which does not inhibit a specific gastroprotective cascade or coprescription with proton pump inhibitors (PPIs) and prostaglandin analogues to suppress acid secretion [12–15]. However, these methods also have limited potency because of their additional cardiovascular effects [16–19].

 Several clinical practice guidelines have proposed different approaches for controlling the GI complications associated with NSAIDs. A number of strategies have been recommended by American College of Gastroenterology to decrease NSAID-induced GI damage including use of selective cyclooxygenase-2 inhibitors, coadministration of gastroprotective agents like misoprostol, PPIs, or histamine-2 receptor antagonists [20]. These strategies are based on multiple risk factors associated with NSAID-induced GI complications including age of the patient, simultaneous medications, prior medical history, and *Helicobacter pylori* infection. The risk of GI bleeding enhances when patients already on antiplatelet therapy using thienopyridines, like clopidogrel, are coprescribed with NSAIDs to reduce adverse cardiovascular events [21]. In 2008, the Clinical Expert Consensus Document prepared by the American College of Cardiology, American College of Gastroenterology and American Heart Association has set the guidelines for reducing GI injury in patients undergoing antiplatelet therapy along with NSAIDs [22]. As per the guidelines, PPIs were recommended for gastroprotective therapy to the patients on thienopyridines and NSAIDs. However, based on some reports suggesting possible interactions between PPIs and thienopyridines [23, 24], the expert guidelines have been further updated in 2010 [25]. The Expert Consensus Report has been prepared taking into account the potential risks and benefits from simultaneous intake of PPIs and thienopyridines. Prescription of PPIs is only recommended for patients on antiplatelet therapy who are at risk for gastrointestinal complications [25].

Till now, there is no effective treatment yet developed for addressing the NSAID-related gastric damage. Identification of the protective factors for gastrointestinal complications associated with NSAIDs still poses a serious challenge. This paper outlines the mechanism of NSAIDs action with their prevalent side effects and provides an insight into the new advances in rational use of NSAIDs for prevention of possible side effects without any compromise on the analgesic properties of the NSAIDs.

## 2. Properties of NSAIDs

NSAIDs possess certain common pharmacologic properties. Mostly they are organic acids with pKa in the range of 3–5 [5]. In general, they contain an acidic group mostly carboxylic acids or enols. The acidic moiety is essential for COX inhibitory activity and is linked to a planar, aromatic group. The latter is also connected to a lipophilic part through a polar group. The NSAIDs are classified into different groups based on their chemical structure and mechanism of action (Table 1). NSAIDs are generally chiral molecules (except diclofenac), but mostly a single enantiomer is pharmacologically active [26].

## 3. Mechanism of Anti-Inflammatory Action of NSAIDs

**Figure 1:**
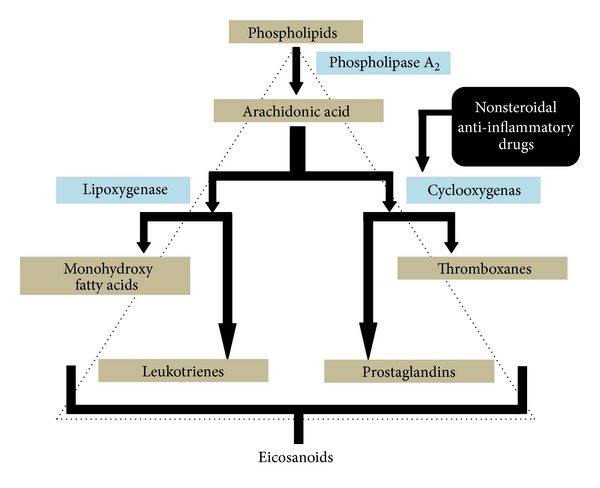
Schematic representation of inhibition of prostaglandin synthesis by NSAIDs.

 The mechanism of action of NSAIDs was first defined in early seventies and is based on inhibition of prostaglandin (PG) synthesis [8]. PG is one of the main mediators of inflammation, pain, and fever and is synthesized from arachidonic acid. The reaction is catalyzed by the enzyme, cyclooxygenase (COX) earlier referred to as PGH synthase [5]. NSAIDs block PG formation by binding and inhibiting COX (Figure 1).

 The analgesic activity of the NSAIDs has been demonstrated to be due to the interference of PGE1 and PGF2 in animal pain models [27, 28]. It has also been observed that NSAIDs are effective against pain because of their ability to inhibit PG-mediated cerebral vascular vasodilation [29, 30]. Several studies have shown that the antipyretic action of NSAIDs is via inhibition of PGE2 synthesis in and near the preoptic hypothalamic area in circumventricular organs [31–33].

## 4. Mechanism of NSAID-Induced GI Injury

**Figure 2:**
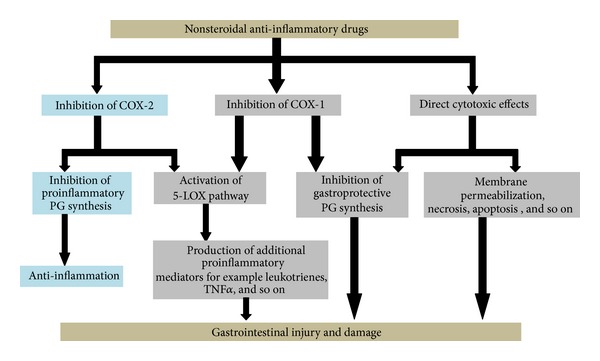
Schematic diagram of mechanism of NSAID-induced gastrointestinal injury and damage.

There are mainly three different mechanisms of NSAID-induced GI complications: inhibition of enzyme COX-1 and gastroprotective PG, membrane permeabilization, and production of additional proinflammatory mediators (Figure 2). 

### 4.1. Inhibition of COX-1 and Gastroprotective PG

There are two isoforms of COX, COX-1 and COX-2, which have different functions [34]. COX-1 is constitutively expressed and is responsible for the normal physiological protection of gastric mucosa. It is responsible for the synthesis of prostaglandins, which protects the stomach lining from the secreted acid, maintains blood flow in gastric mucosa, and produces bicarbonate [35, 36]. The other isoform, COX-2, is triggered by cell damage, various proinflammatory cytokines, and tumor-derived factors [37, 38]. NSAID-induced gastropathy is caused mainly by inhibition of COX-1 by NSAIDs [39–41]. 

### 4.2. Membrane Permeabilization

NSAIDs also have a direct cytotoxic effect on gastric mucosal cell causing lesions and injury [42, 43]. Some studies have shown that direct cytotoxicity is independent of the inhibition of COX activity [44]. Topical damage of this kind has been observed in the case of acidic NSAIDs like aspirin resulting in accumulation of ionized NSAID, a phenomenon called “ion trapping” [45]. It is suggested that NSAIDs cause membrane permeabilization leading to disruption of epithelial barrier [46]. NSAIDs were also able to induce both necrosis and apoptosis in gastric mucosal cells [47].

### 4.3. Production of Additional Proinflammatory Mediators

Inhibition of PG synthesis by NSAIDs leads to simultaneous activation of the lipoxygenase pathway and increased synthesis of leukotrienes (Figure 1) [48–50]. Leukotrienes cause inflammation and tissue ischaemia leading to gastric mucosal injury [51, 52]. Along with this, there is also enhanced production of proinflammatory mediators such as tumour necrosis factors [53]. This further leads to occlusion of gastric microvessels leading to reduced gastric blood flow and release of oxygen-derived-free radicals [54]. Free oxygen radicals react with poly unsaturated fatty acids of the mucosa leading to lipid peroxidation and tissue damage [54].

## 5. Current Therapies for Prevention of Gastric Damage

Several approaches have been adopted for addressing the prevention and cure of the possible side-effects produced by the NSAIDs in the gut. Some of these strategies are routinely prescribed to the patients administering NSAIDs. Presently, the most common protective strategies adopted are (1) combination therapy of NSAIDs with gastroprotective agents and (2) use of selective COX-2 inhibitors (Table 2).

### 5.1. Combination Therapy of NSAIDs with Gastroprotective Agents

#### 5.1.1. PG Analogues

PG analogues are prescribed with NSAIDs for replenishment of PG which is inhibited by NSAIDs. Misoprostol, a widely used PG analogue, was found to reduce NSAID-induced gastroduodenal ulceration considerably [12]. However, it fails to prevent the reduction of dyspepsia and other GI adverse effects and hence has a limited efficiency [55, 56]. Recently it has been reported that the single-tablet formulations of diclofenac and misoprostol which have been found to be effective in arthritis and in reducing the NSAID-induced gastropathy [57].

#### 5.1.2. Acid Suppressants

Acid increases NSAID-induced mucosal injury and gastric absorption of acidic NSAIDs. H2-receptor antagonists and proton pump inhibitors (PPIs) are most commonly used because they not only reduce acid secretion but also enhance gastric pH and have a role in scavenging-free radicals [58, 59].

H2-receptor antagonists were the first drugs to be used as a prevention mechanism against NSAID-induced peptic ulcers [60]. They were found to be effective against gastric ulceration to a considerable extent [61]. However, no signs of improvement were observed in cases of gastric bleeding, [62] and hence, these drugs are no longer recommended presently. 

 PPIs are effective in terms of acid suppression and prevention of peptic ulcers when coadministered with the NSAIDs. PPIs are generally prescribed for long-term use since they do not show any significant risk of any associated effects [63, 64]. Omeprazole, a PPI widely prescribed with NSAIDs, can specifically block the parietal cell H^+^/K^+^-ATPase, thereby significantly inhibiting the gastric acid secretion [65]. Omeprazole was followed by other PPIs like lansoprazole, pantoprazole, rabeprazole, and so forth [66]. Another report has indicated the formulation of lansoprazole, in the form of fast disintegrating tablet to reduce GI injury [67]. Esomeprazole, the S-isomer of omeprazole, has been found to provide a sustained gastric acid control as compared to other PPIs [68]. Considerable reduction of adverse GI symptoms has been observed in patients prescribed with esomeprazole along with NSAIDs or selective COX-2 inhibitors [69, 70]. The first NSAID/PPI single tablet formulation to be approved is ketoprofen/omeprazole modified release capsules [71].

Dual antiplatelet therapy with thienopyridine like clopidogrel and NSAID like aspirin is prescribed to decrease adverse cardiac events in patients suffering from acute coronary syndromes or placement of an intracoronary stent [72, 73], but they are associated with high risks of GI bleeding [21]. PPIs are found to be effective in reducing the risk of GI bleeding in such patients [23]. Clopidogrel is a prodrug that is transformed in vivo to an active metabolite by the cytochrome P450 enzyme system [74]. However, some reports have suggested that PPIs interfere with clopidogrel to impair platelet function [23, 24, 75]. PPIs possibly inhibit hepatic cytochrome P450 2C19 (CYP2C19) isoenzyme preventing the conversion of clopidogrel into its active metabolite. It has been reported that concurrent use of clopidogrel plus a PPI was associated with a significant increase in risk of an adverse cardiovascular event in patients with acute chronic syndrome [76, 77]. In contrast to this, some other trials did not find any enhanced risk of adverse effects of the use of PPI in combination with clopidogrel [78, 79]. Thus, though routine use of a PPI is not recommended for patients in general, but it is coprescribed in patients with potential risk of GI bleeding [25, 80].

The main drawback of PPIs is that they are less effective against mucosal injury in more distal parts of the intestine like NSAID-induced colonopathy [81]. Moreover, these agents are not prescribed to patients suffering from *H. pylori* infection because of occurrence of corpus gastritis [82].

### 5.2. Selective COX-2 Inhibitors

Selective COX-2 inhibitors, as the name suggests, are a group of drugs which selectively inhibit the COX-2 inhibitors, thus maintaining the anti-inflammatory properties of NSAIDs, yet retaining the gastroprotective action elicited by COX-1 pathway [83–85]. By far, celecoxib and rofecoxib stand out as the most effective COX-2 inhibitors and show efficacy over nonselective NSAIDs in regard to GI complications including mucosal lesions and other adverse GI symptoms [86, 87]. 

 Several classes of COX-2-selective inhibitors have been identified, including the diarylheterocyclics (or tricyclics), acidic sulfonamides, and 2,6-ditert-butyl phenols, as well as the derivatives of the nonselective inhibitors zomepirac, indomethacin, piroxicam, and aspirin [88–90]. Celecoxib was first identified in 1997 and approved in 1998 [91, 92]. It has been found to preferentially inhibit COX-2 but exhibited the anti-inflammatory, antipyretic, and analgesic activities of NSAIDs [86, 93, 94]. Rofecoxib launched in 1999 was found to be effective in the treatment of osteoarthritis and pain [87, 95–97]. Similarly, nimesulide was highly selective against COX-2, so that at concentrations attained in vivo, while it had no substantial effect on COX-1, it suppressed COX-2 significantly [98]. 

 Though COX-2 inhibitors decrease the GI toxicity to a considerable amount, there is an associated risk of cardiovascular complications due to myocardial infarction and thrombosis associated with their use [99–104]. COX-2 inhibitors have been demonstrated to inhibit the production of vascular prostacyclin, which has vasodilatory effects, and inhibits platelet aggregation unlike nonselective NSAIDs [105, 106]. Longer term gastrointestinal data from the celecoxib study (CLASS) and cardiovascular adverse event data from the rofecoxib study (VIGOR) have questioned the usage of these new drugs [86, 87, 107]. Some of these potent drugs have even been withdrawn [108].

## 6. Recent Advances in NSAID Treatments 

### 6.1. Prodrugs of NSAIDs

NSAID prodrugs are potential agents for enhancing the antioxidant activity, water solubility and dissolution, release of nitric oxide and hydrogen sulfide, site-specific targeting and delivery, and inhibiting anticholinergic and acetylcholinesterase activity [109–113]. 

#### 6.1.1. Nitric Oxide Releasing NSAIDs

It has been observed that nitric oxide (NO) imparts gastroprotection by increasing blood flow, mucus production, and bicarbonate secretion in the gastric mucosa [114–116]. NO formed by the action of nitric oxide synthase increases mucus and bicarbonate secretion as well as microcirculation and decreases neutrophil-endothelial adherence [117]. This led to the development of new therapeutic drugs: nitric oxide releasing NSAIDs (NO-NSAIDs) [118]. These drugs are developed by modifying NSAIDs esterified to a NO releasing moiety. Animal studies have demonstrated that NO-NSAIDs do not affect the gastroduodenal mucosa [119–121]. NO naproxen has been also been found to enhance anti-inflammatory and antinociceptive efficacy [122]. NO aspirin has been found to impart an increased antithrombotic potency compared with conventional aspirin [123, 124]. 

#### 6.1.2. Hydrogen Sulfide Releasing NSAID

Hydrogen sulfide (H2S) also exerts its gastroprotective effects and reverses preexisting ulcers. Derivatives of naproxen, diclofenac, and indomethacin which can release H2S have been reported [125–128]. Phosphatidylcholine-associated NSAIDs as well as NO- and H2S-releasing NSAIDs are under extensive preclinical testing for their influence on NSAID induced GI toxicity [129, 130].

Further studies are in progress to develop promising new NSAIDs imparting total GI (upper and lower GI tracts) protection and without cardiovascular toxicity. Recently a diclofenac prodrug, 1-(2,6-dichlorophenyl)indolin-2-one, has been demonstrated with anti-inflammatory properties that can decrease PGE2 levels, COX-2 expression, and ulceration [131]. In yet another experiment, it was observed that ibuprofen R(−) isomer is a better agent in preventing GI toxicity than S(+) isomer because of short plasma-elimination half-life, its limited ability to inhibit PG synthesis. The R(−) isomer is then converted in the body to the S(+) isomer after absorption in the GI tract [132]. 

### 6.2. Simultaneous Inhibition of COX and 5-LOX

NSAID-induced inhibition of COX also results in increased production of leukotrienes, one of the potent mediators of inflammation [49–51]. Recent approach for addressing NSAID-induced GI injury is by development of inhibitors of COX/5-LOX simultaneously [133, 134]. Licofelone ([2,2-dimethyl-6-(4-chloropheny-7-phenyl-2,3-dihydro-1H-pyrrazoline-5-yl]acetic acid) has been identified as one of the most convincing compounds in this group [135]. Licofelone imparts significant analgesic and anti-inflammatory effects without any GI side-effects as observed in animal models [136]. It significantly improved indomethacin-induced gastric ulceration and prevented NSAID-induced increase in leukotriene levels in gastric mucosa [137]. The preclinical evaluation has suggested that licofelone has a promising pharmacodynamic effect [138]. Further clinical trials are in progress in osteoarthritis patients [139]. Licofelone has also been found to be effective because of its antithrombotic and platelet aggregation inhibiting functions [140]. Earlier to this, benoxaprofen identified as a dual COX/5-LOX inhibitor was withdrawn because it was found to induce severe hepatic and other toxicities [141].

### 6.3. Role of Lactoferrin in Reducing NSAID-Induced Gut Damage

Some preliminary reports have shown that bovine colostrum has the ability to prevent NSAID-induced gastric ulcers [142, 143]. Further studies have demonstrated the role of recombinant human lactoferrin in decreasing acute NSAID-induced GI bleeding and reduction of gastric ulcers [144, 145]. Recent reports also suggest that C-lobe of lactoferrin, which is resistant to enzymatic degradation [146], has excellent sequestering property for such class of drugs [147]. Further reports have shown that C-lobe of lactoferrin can also bind to COX-2-specific drugs and produce observable effects against gastric inflammation and bleeding [148]. Experiments on rodent model suggest that C-lobe of lactoferrin considerably diminishes the NSAID-induced GI bleeding and inflammation in case of conventional NSAIDs as well as COX-2-specific NSAIDs [147]. In this regard, development of such new molecules that can sequester the unbound drug molecules is essential for addressing the NSAID-related GI damage.

## 7. Conclusions

The therapeutic effects of NSAIDs have made these drugs extremely popular against inflammatory disorders for the past several decades. However, these drugs suffer from serious drawbacks in cases of long-term administration, including severe GI complications. Several strategies have been adapted to control the critical side-effects. Though, these treatments are effective to some extent, but most of them are also associated with other risks.

Thus, there is no drug yet formulated that can avert the potential side-effects completely. There is an urgent need to develop novel therapeutic agents to make the use of NSAIDs safer. New measures of treatments such as dual COX/5-LOX inhibitors, prodrugs of NSAIDs, or agents that can effectively sequester the unbound NSAIDs without interfering their efficacy can prove to be superior strategies compared to the existing ones.

## Figures and Tables

**Table 1:** Classification of NSAIDs.

Types	Chemical composition	Common NSAIDs
Salicylates	Derivatives of 2-hydroxybenzoic acid (salicylic acid)	Aspirin, diflunisal, and salsalate

Propionic acid derivatives or “profens”	Derivatives of arylacetic acids	Ibuprofen, dexibuprofen, ketoprofen, dexketoprofen, naproxen, fenoprofen, flurbiprofen, oxaprozin, and loxoprofen

Acetic acid derivatives	Derivatives of acetic acids	Indomethacin, diclofenac, nabumetone, tolmetin, sulindac, etodolac, and ketorolac

Enolic acid derivatives or oxicams	Derivatives of 4-hydroxy benzothiazine heterocycle	Piroxicam, isoxicam, meloxicam, tenoxicam, droxicam, and lornoxicam

Fenamic acid derivatives or fenamates	Derivatives of anthranilic acid	Mefenamic acid, flufenamic acid, tolfenamic acid, and meclofenamic acid

Phenylpyrazolones	Derivatives of 1-aryl-3,5- pyrazolidinedione	Phenylbutazone, oxyphenbutazone

COX-2 selective inhibitors	Diaryl-5-membered heterocycles	Celecoxib, rofecoxib, and valdecoxib

Anilides and sulphoanilides	Acetamides of aniline with or without a 4-hydroxy or 4-alkoxy group	Acetaminophen, phenacetin, and nimesulide

**Table 2:** . Strategies to prevent NSAID-induced gastrointestinal injury.

Treatment procedure	Mechanism	Action
Gastroprotective drugs		
(i) PG analogues	Replacement of PG	Reduces ulceration and other GI damages Cannot prevent dyspepsia
(ii) Acid suppressants like proton pump inhibitors	Increase of intragastric pH	Decreases dyspepsia, ulceration, and associated damages Not suitable for patients with *H. pylori* infections

Selective COX-2 inhibitors	Does not inhibit COX-1, and hence synthesis of gastroprotective PG is maintained	Reduces dyspepsia, reverses gastroduodenal ulcers, and prevents other GI damages Associated with prothrombotic events and enhances cardiovascular risks

NSAID prodrugs like NO-NSAIDs	Release of NO maintains microvascular integrity	Reduces GI damage, has antithrombotic effects

Inhibitors of COX and 5-LOX	Blocks formation of leukotrienes and other proinflammatory mediators	Maintains gastroprotection and reduces GI damage

Role of lactoferrin	Structural studies suggest binding of C-terminal lobe of lactoferrin with NSAIDs and sequestration of unwanted NSAIDs	Animal studies indicate reversal of gastric bleeding and inhibition of myeloperoxidase formation
